# Successful fluconazole combined with caspofungin treatment of candida bloodstream infection in preterm infant: A case report

**DOI:** 10.1097/MD.0000000000028270

**Published:** 2021-12-30

**Authors:** Gaole Yuan, Yingqiu Tu, Lili Liu, Tiantian Xu

**Affiliations:** The First Affiliated Hospital of Nanchang University, Nanchang, Jiangxi China.

**Keywords:** candida bloodstream infection, caspofungin, fluconazole, preterm infant

## Abstract

**Rationale:**

Candida bloodstream infection continues to be a significant cause of mortality in premature infants. Amphotericin B has been recommended as the primary treatment; however, its use is limited due to drug-induced nephrotoxicity and amphotericin B-resistant candidemia.

**Patient concerns:**

The gestational age was 29 (+6) weeks, and birth weight was 1760 g.

**Diagnosis:**

The infant was diagnosed with Candida parapsilosis bloodstream infection.

**Interventions:**

Fluconazole, 12 mg/kg/day, combined with caspofungin (loading dose 3 mg/kg, at a maintenance dose of 2 mg/kg every 24 h) therapy was administered to premature infant with Candida bloodstream infection. When fluconazole or caspofungin was used to treat Candida bloodstream infection in preterm infants, the blood cultures of the infant remained positive for Candida parapsilosis.

**Outcomes:**

All persistent candidemia resolved on fluconazole combined with caspofungin therapy. There were no adverse effects, hepatotoxicity, nephrotoxicity, anemia, or thrombocytopenia.

**Lessons:**

Fluconazole combined with caspofungin successfully treated Candida bloodstream infection in premature infants at 29 + 6 weeks’ gestational age, but large-scale clinical trials are required.

## 1 Introduction

Candida is an important pathogen of neonatal infections and represents the third most common causative agent of late-onset sepsis, and has a high burden of morbidity and mortality.^[1]^ The majority of fungal infections are caused by Candida albicans and Candida parapsilosis.^[2]^ Candida infection occurs in 4% to 18% of critically ill neonates, with a higher incidence among extremely low birth weight infants (birth weight≤1000 g).^[3]^ Fluconazole and amphotericin B are the preferred treatments for neonatal invasive candidiasis and candidemia. We report a case of fluconazole combined with caspofungin for successful therapy of Candida parapsilosis in preterm infants. The successful treatment of fluconazole with caspofungin for neonatal Candida parapsilosis bloodstream infection was first reported.

## 2 Case

A male infant was born at 29 + 6 weeks of gestational age by natural labor, with a birth weight of 1760 g and Apgar score of 7 to 8 at 1 and 5 minutes. Total parenteral nutrition was infused because of enteral feeding intolerance. Broad-spectrum antibiotic coverage with penicillin and cefoperadone sodium sulbactam sodium was administered. At 5 days, a peripherally inserted central catheter (PICC) was placed. At 29 days, the infant presented with scattered floral markings, low muscle tension, and signs of catheter-associated bloodstream infection. Serum procalcitonin level was 0.5ng/mL, and serum C-reactive protein were 14.28 mg/L. Fluconazole (6 mg/kg every 24 hours) was promptly administered. At 31 days, blood cultures were positive for fungi, and the tip of the PICC was cultured positive for fungi. Then, PICC was removed, and fluconazole was added to12 mg/kg/d. At 33 days, the drug sensitivity results were reported for Candida parapsilosis, sensitive to fluconazole and caspofungin; therefore, an adequate amount of fluconazole was continued for 14 days. At 48 days, the infant had a fever, and blood culture was positive for Candida parapsilosis, sensitive to fluconazole and caspofungin. Serum procalcitonin level was added to 7.73 ng/mL, while serum C-reactive protein was added to 30.06 mg/L. Fluconazole was stopped at this time and replaced with caspofungin (loading dose 3 mg/kg, at a maintenance dose of 2 mg/kg every 24 hours) for 14 days. However, at 68 days, blood culture was positive for Candida parapsilosis and sensitive to fluconazole and caspofungin; therefore, caspofungin (2 mg/kg every 24 hours) was used in combination with fluconazole. At 77 days, the blood culture was cleared, and the clinical condition improved. There were no adverse effects, hepatotoxicity, nephrotoxicity, anemia, or thrombocytopenia.

## 3 Discussion

In this case, the infant presented multiple risk factors for candidiasis, such as low gestational age, central venous line placement, lack of enteral feeding, total parenteral nutrition with lipid infusion, and broad-spectrum antibiotic exposure. The infant was treated with adequate fluconazole for 14 days; however, it was not effective. However, there are still variations in practice regarding the use of fluconazole for the prevention and treatment of invasive candidiasis.^[4]^ Therefore, caspofungin was replaced with fluconazole in the treatment of neonatal Candida for 14 days. However, at this time, the total serum bilirubin and direct bilirubin levels continued to rise, considering that it was caused by a Candida infection. Later, fluconazole was combined with caspofungin to achieve a treatment effect. The total serum bilirubin and direct bilirubin levels returned to normal levels. Fluconazole, 12 mg/kg intravenous or oral daily, is a reasonable alternative in patients who have not received fluconazole prophylaxis.^[5]^ The infant used fluconazole to prevent Candida infection after birth; therefore, fluconazole treatment for neonatal candidiasis is not the best choice. At that time, the hospital did not receive amphotericin B. Therefore, echinocandins have been used to treat neonatal candidiasis. Echinocandins should be used with caution and are generally limited to salvage therapy or to situations in which resistance or toxicity precludes the use of AmB deoxycholate or fluconazole.[^5^,^6^] Among the echinocandins, micafungin is the most studied echinocandin in neonates and is only approved by both the European Medicine Agency and the United States Food and Drug Administration for younger children.[^7^,^8^,^9^] Caspofungin is FDA approved in children >3 months of age and European Medicine Agency approved in children >12 months of age to treat presumed fungal infections in febrile, neutropenic patients, candidemia, and invasive aspergillosis in patients refractory to or intolerant of other therapies.[^10^,^11^] However, among neonates and infants with confirmed invasive Candida infection, fungal-free survival at 2 weeks was similar in the caspofungin and amphotericin B deoxycholate treatment arms. A smaller proportion of participants who received caspofungin experienced adverse events.^[12]^ To date, efficacy studies of caspofungin have not been conducted in the neonatal population. Clinical trials in a larger neonatal population are needed in the future.^[13]^

No comparative studies of fluconazole with other agents such as echinocandins or other triazole antifungal agents for the treatment of neonatal candidiasis are available.^[4]^ The successful treatment of fluconazole with caspofungin for neonatal Candida parapsilosis bloodstream infection was first reported.

## Author contributions

**Data curation:** Gaole Yuan, Yingqiu Tu.

**Formal analysis:** Tiantian Xu.

**Investigation:** Gaole Yuan, Lili Liu.

**Writing – original draft:** Yingqiu Tu.

**Writing – review & editing:** Yingqiu Tu.
